# Electroacupuncture Alleviates Comorbid Depression‐Like Behaviors and Dyspepsia‐Like Symptoms in Rats Accompanied by Alterations in Hippocampal Neuroinflammation and Gut Microbiota

**DOI:** 10.1002/brb3.71854

**Published:** 2026-10-22

**Authors:** Tianlang Wang, Yifan Feng, Xihan Wang, Yanan Xue, Jianbo Wang, Yi Qu, Tieming Ma, Jidong Liu

**Affiliations:** ^1^ College of Acupuncture and Tuina Liaoning University of Traditional Chinese Medicine Shenyang Liaoning China; ^2^ Liaoning University of Traditional Chinese Medicine Shenyang Liaoning China

**Keywords:** brain–gut microbiota axis, comorbid depression and dyspepsia, electroacupuncture, hippocampal microglia, mtROS‐NLRP3 signaling

## Abstract

**Background:**

Depression and dyspepsia are highly comorbid in clinical practice, and their occurrence and progression are closely associated with dysfunction of the brain–gut axis. Electroacupuncture (EA) can improve depression and gastrointestinal dysfunction at the same time. However, whether its therapeutic effects are mediated through regulation of the brain–gut axis remains unclear.

**Methods:**

We established a rat model of comorbid depression‐like and dyspepsia‐like symptoms using SD rats. Behavioral tests were used to evaluate anxiety‐ and depression‐like states in rats. Gastric emptying rate, small intestinal propulsion rate, and gastrointestinal hormones were measured to assess gastrointestinal motility. Histomorphological changes in the hippocampus, stomach, and duodenum were observed by hematoxylin–eosin (HE) staining and Nissl staining. Immunofluorescence (IF), Western blotting, quantitative real‐time polymerase chain reaction (RT‐qPCR), and biochemical assays were used to determine the activation status of hippocampal microglia and mitochondrial reactive oxygen species (mtROS)/NOD‐like receptor family pyrin domain containing 3 (mtROS/NLRP3) pathway‐related indicators. Enzyme‐linked immunosorbent assay was used to determine central and peripheral levels of 5‐hydroxytryptamine (5‐HT) and inflammatory factors. Gut microbiota diversity and composition were analyzed by 16S rRNA sequencing. The expression of duodenal mucosal barrier‐related proteins was detected by immunofluorescence, and the ultrastructure of the duodenum was observed by transmission electron microscopy.

**Results:**

EA significantly alleviated anxiety‐ and depression‐like behaviors and improved gastrointestinal motility in model rats. EA ameliorated hippocampal neuronal injury, increased Nissl body number, reduced mtROS and malondialdehyde levels, enhanced mitochondrial membrane potential and superoxide dismutase activity, and suppressed microglial activation and NLRP3 inflammasome signaling. In addition, EA increased central and peripheral 5‐HT levels while decreasing Interleukin‐6 (IL‐6) and tumor necrosis factor‐α expression. EA also modulated gut microbiota composition by reducing opportunistic pathogens and enriching beneficial bacteria. Moreover, EA improved duodenal ultrastructure and upregulated the tight junction proteins ZO‐1 and Occludin.

**Conclusion:**

EA can improve depression‐like behaviors and dyspepsia‐like symptoms in comorbid rats. These behavioral and gastrointestinal improvements were accompanied by reduced hippocampal oxidative stress and neuroinflammation, altered gut microbiota composition, and improved duodenal mucosal barrier. These findings suggest that the therapeutic mechanism of EA may involve regulation of the brain–gut axis, although the specific causal relationships among its individual components remain to be elucidated.

## Graphical abstract

EA improves depression and dyspepsia via brain‐gut axis regulation

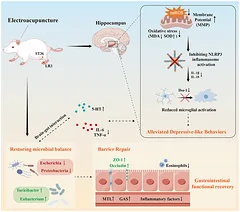

## 1 Introduction

Depression and dyspepsia are two common clinical disorders with high prevalence. Existing studies have demonstrated that they have a remarkably high comorbidity rate (Du et al. 2024; Singh et al. 2022). More than half of patients with dyspepsia present with comorbid depressive or anxiety symptoms, while patients with depression frequently exhibit dyspeptic symptoms such as loss of appetite and early satiety (Esterita et al. 2021). These symptoms may mutually reinforce each other, creating a vicious cycle between emotional disturbance and digestive dysfunction that substantially impairs patients' quality of life. Therefore, clarifying the mechanisms underlying depression–dyspepsia comorbidity and identifying effective therapeutic strategies are of considerable clinical importance.

Accumulating evidence suggests that dysfunction of the brain–gut axis plays an important role in the development of depression comorbid with dyspepsia (Huang et al. 2023). As a key component of the brain–gut axis, the gut microbiota has attracted increasing attention because of its involvement in both gastrointestinal homeostasis and central nervous system function. Gut microbiota dysbiosis may impair intestinal barrier integrity, activate mucosal immune responses, and contribute to visceral hypersensitivity and gastrointestinal motility disorders, thereby promoting dyspeptic symptoms (Tziatzios et al. 2020; Zheng et al. 2025). Meanwhile, microbiota‐derived signals may influence the central nervous system and participate in neuroinflammatory processes associated with mood disorders (Liu et al. 2023). In parallel, chronic stress and negative emotional states can disturb neuroimmune homeostasis and further aggravate gastrointestinal dysfunction and microbial imbalance (Leigh et al. 2023). As a key brain region for emotion regulation, the hippocampus is particularly sensitive to chronic stress. Hippocampal neuronal injury, impaired synaptic plasticity, and neuroinflammation are considered important pathological features of depression (Herzberg et al. 2024; Zhu et al.^2025^). Studies have shown that chronic stress disrupts mitochondrial function in hippocampal neurons, as reflected by a reduction in mitochondrial membrane potential (MMP) and excessive production of mitochondrial reactive oxygen species (mtROS), which impairs neural plasticity (Song et al. 2023; Wang et al. 2025). Modulation of mitochondrial function and attenuation of oxidative stress in hippocampal neurons have been shown to increase excitatory synaptic density, enhance neuronal activity, and alleviate depression‐like behaviors (Zhu et al. 2025). Moreover, abnormal accumulation of mtROS can further activate the NOD‐like receptor family pyrin domain containing 3 (NLRP3) inflammasome, promoting the maturation and release of pro‐inflammatory factors such as interleukin‐1β (IL‐1β) and IL‐18 (Sorbara and Girardin 2011). Activation of the NLRP3 inflammasome is considered a key mechanism in the pathogenesis of depression (Xia et al. 2023). The NLRP3 inflammasome is highly expressed in microglia; its activation induces the transition of microglia from a resting to an activated state, resulting in the release of large amounts of inflammatory factors (Gao et al. 2023). This process sustains inflammation within the hippocampal microenvironment and further accelerates the progression of depression (Wang et al. 2021).

Current treatment of depression accompanied by dyspeptic symptoms often involves combinations of antidepressants, prokinetic agents, or probiotics (Gastaldo‐Jordán et al. 2025). However, the long‐term efficacy of these treatments remains limited, and some antidepressants may produce gastrointestinal adverse effects that further aggravate digestive symptoms (Oliva et al. 2021; Wen et al. 2025). Therefore, it is essential to explore multi‐targeted, safe, and effective alternative therapies. Acupuncture has shown therapeutic potential in both emotional disorders and gastrointestinal dysfunction (Chen et al. 2025; Xu et al. 2024). Previous studies have demonstrated that electroacupuncture (EA) exerts antidepressant effects by inhibiting neuroinflammation and regulating gut microbiota (Zeng et al. 2025). In this study, Zusanli (ST36) and Taichong (LR3) were selected as the intervention acupoints. LR3 is a key acupoint for the treatment of depression (Tu et al. 2023) and has been reported to regulate the function of emotion‐related brain regions, including the prefrontal cortex and hippocampus (Yang et al.^2023^; Zhang et al. 2024). It is also a primary acupoint for managing gastrointestinal disorders (Moon et al. 2023). EA at ST36 can not only exert antidepressant effects by restoring the neuronal structure and morphology of the hippocampal CA1 area and increasing the expression of brain‐derived neurotrophic factor (Wang et al.^2025^), but also correct the disorder of gut microbiota, increase the abundance of beneficial bacteria to improve symptoms of dyspepsia, and modulate neurotransmitter levels to alleviate depressive symptoms (Wang et al. 2024).

Although the beneficial effects of EA in depression or gastrointestinal disorders have been increasingly reported, evidence regarding its role in depression–dyspepsia comorbidity remains limited. It is still unclear whether the effects of EA on this comorbid condition are accompanied by changes in gut microbiota composition and hippocampal neuroinflammation‐related indices. Therefore, in this study, a rat model of depression‐like and dyspepsia‐like comorbidity was established to systematically evaluate the effects of EA on behavioral abnormalities and gastrointestinal dysfunction and to further explore its potential mechanisms.

## 2 Materials and Methods

### 2.1 Animals

Thirty 4‐day‐old healthy male SPF‐grade SD young rats (weight 5–8 g) and three 10‐week‐old SPF‐grade female SD rats (weight 250–300 g) were purchased from Liaoning Changsheng Biotechnology Co., Ltd. (Laboratory animal feeding license No. SCXK (Liaoning) 2020‐0001), and the feeding environment was an independent cage in the Animal Experiment Center of Liaoning University of Traditional Chinese Medicine. The rearing environment was clean and well‐ventilated; the room temperature was maintained at 20°C–25°C, the relative humidity was 45%–55%, and the light–dark cycle was 12 h/12 h. This experiment was approved by the ethical review of the Animal Experiment Center of Liaoning University of Traditional Chinese Medicine (Ethical Review Approval No. 21000062024086), and the experimental process strictly followed the ethical norms of animal experimentation for animal handling.

### 2.2 Model Establishment and Animal Grouping

Four‐day‐old rats were randomly assigned to three groups: (1) control group (*n* = 10), (2) model group (*n* = 10), and (3) EA group (*n* = 10). After 3 days of breastfeeding, the model group and the EA group received iodoacetamide by gavage and chronic unpredictable mild stress (CUMS) intervention (Antoniuk et al. 2019; Wang et al. 2025). Rats in the control group received 2% sucrose solution, whereas those in the model group and EA group received 2% sucrose solution containing 0.1% iodoacetamide by gavage, 0.2 mL per rat, once a day for 7 days. This was followed by a 2‐week period of adaptive feeding. When the rats were 4 weeks old, except for the control group, the other two groups received CUMS stimulation. The CUMS procedures included (1) food deprivation for 24 h; (2) water deprivation for 12 h; (3) forced swimming in ice water for 10 min; (4) standing on a small platform in a water environment for 12 h; (5) cage tilting for 12 h; (6) noise stimulation for 3 h; (7) wet cage rearing for 12 h; and (8) cage shaking for 5 min. Two stressors were randomly applied each day, with no identical stimulation schedule used on two consecutive days. The modeling procedure lasted for 28 days, during which the general condition of the rats was monitored.

### 2.3 EA Intervention

The rats in the EA group were secured in a self‐made restraining device in the prone position. Intervention was carried out using disposable sterile acupuncture needles (0.25 mm × 13 mm, from Suzhou Medical Device Factory Co., Ltd.). Acupoint locations were determined according to the rat acupoint standards in Experimental Acupuncture (Yu and Guo 2009), and bilateral Zusanli (ST36) and Taichong (LR3) were selected. ST36 is located on the anterior side of the lower leg, 5 millimeters below the lateral edge of the patella, between the anterior tibialis muscle and the extensor digitorum longus muscle, with a needle depth of 5 mm. LR3 is located in the depression in front of the junction of the base of the first and second metatarsal bones on the dorsum of the foot, with a needle puncture depth of 2 mm. After disinfection of the acupoints, acupuncture is performed, and the EA device (SDZ‐III; Suzhou Medical Device Factory Co., Ltd.) is connected. Each pair of electrodes is connected to ST36 and LR3 on the same side, with one pair on each side. The current intensity was determined when the hind limbs of the rats showed slight tremors but no restlessness or struggle. The waveform was selected as dense‐sparse waves, and the frequency was set at 2/10 Hz (determined through previous screening). The intervention was carried out once a day, each time for 20 min, for 14 consecutive days. During this period, the condition of the rats needs to be closely observed to ensure that the operation does not cause excessive stress to the animals. The overall experimental process is shown in Figure 1A.

**FIGURE 1:**
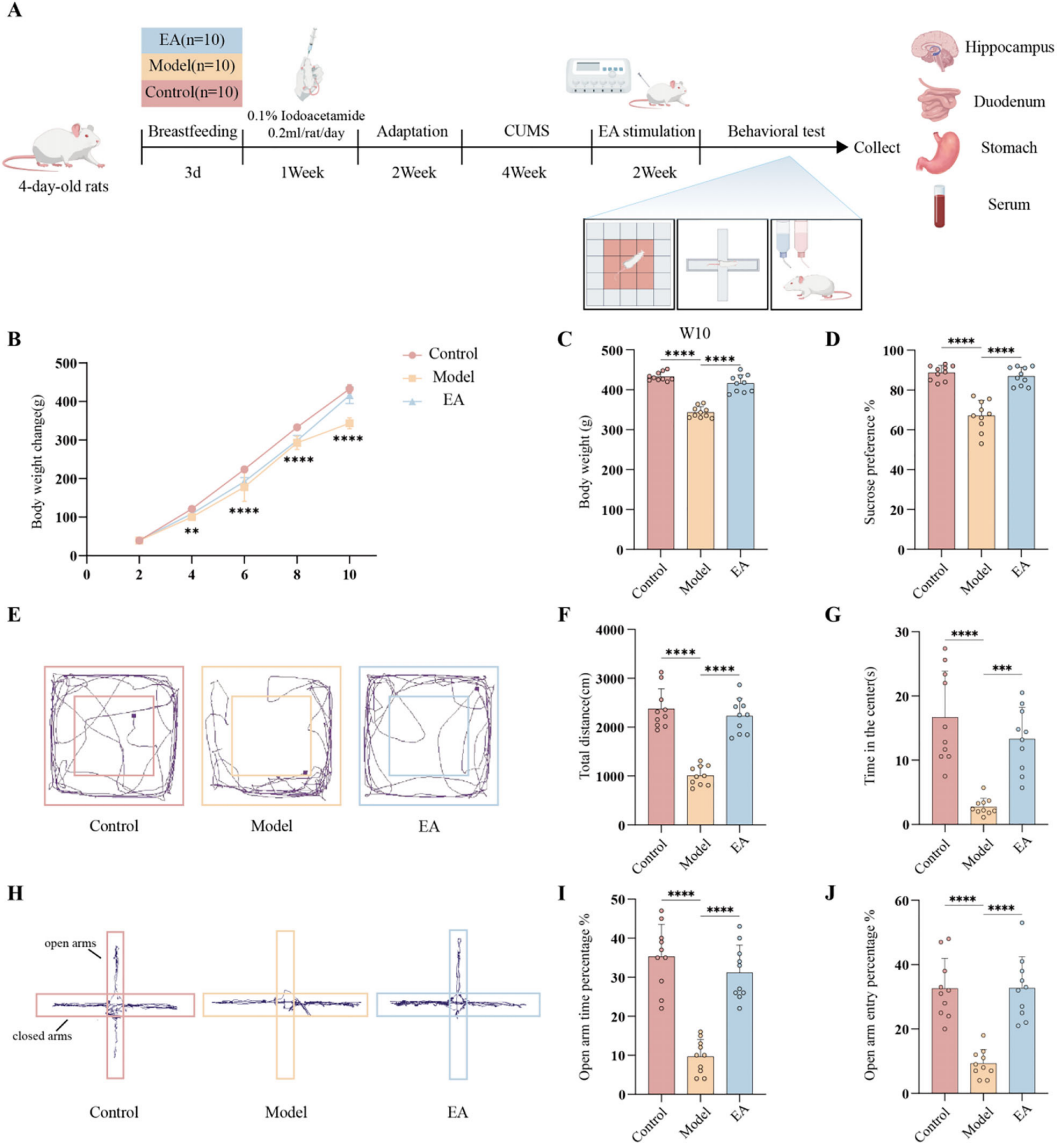
Overall experimental process, weight measurement, and behavioral tests. (A) Overall process description of the experiment. (B, C) The overall weight change trend and the body weight at the 10th week. At each time point, *n* = 10 for each group. ^**^ *p* < 0.01, ^****^ *p* < 0.0001 vs. control group. (D) Sucrose preference in SPT. (E–G) The motion trajectory, total movement distance, and central residence time in OFT. (H–J) The motion trajectory in EPM, the percentage of times entering the open arm, and the percentage of time staying in the open arm. Data are expressed as mean ± SD. ^**^ *p* < 0.01, ^***^ *p* < 0.001, ^****^ *p* < 0.0001. *n* = 10 per group.

### 2.4 Behavioral Testing

#### 2.4.1 Open Field Test (OFT)

One hour before the test, the rats were transferred to the laboratory to acclimatize to the environment. The test was conducted in an open field box measuring 100 cm × 100 cm × 40 cm, with the central area being 50 cm × 50 cm (Knight et al. 2021). The rats were allowed to explore freely for 5 min. Following the acclimatization period, each rat was positioned at the central starting point. The experimenter then moved to a location 1 m away from the observation area to record a video capturing the 5‐min activity trajectory of the rats. After testing each rat, the box was cleaned with 75% ethanol and allowed to air‐dry naturally to eliminate any residual odors. Finally, a video tracking system was used to analyze the total movement distance of each rat and the time spent in the central area.

#### 2.4.2 Elevated Plus Maze (EPM)

The EPM consisted of two open arms and two closed arms. The open arms and closed arms were arranged in a cross pattern, and the central platform area was 10 cm × 10 cm (Kraeuter et al. 2019). Rats were allowed to freely explore for 5 min before the experiment. At the beginning of the experiment, the rats were placed on a central platform with their heads facing open arms. The experimenters immediately left and recorded the rats' activities for 5 min through a camera. After each rat was tested, the maze was thoroughly cleaned with 75% ethanol to eliminate any residual odors. The number of times rats entered the open arm and the closed arm and the duration of their stay were analyzed using video tracking software, and the percentages of the number of entries into the open arm and the duration of stay in the open arm were calculated.

#### 2.4.3 Sucrose Preference Test (SPT)

Anhedonia behavior in rats was evaluated using the sucrose preference test (SPT) to determine the depression‐like state of rats (Liu et al. 2018). The experiment was divided into two stages: the training and testing periods. The training period lasted for 48 h. During this period, two bottles of liquid were placed simultaneously in the cage, one containing 1% sucrose water and the other containing pure water. The positions of the two bottles were swapped every 12 h to avoid positional preference. After training was completed, a 24‐hour test period was entered. During this period, rats were deprived of food and water. One bottle of 1% sucrose water and one bottle of pure water were provided simultaneously. The positions were swapped every 6 h, and the consumption of the two bottles of liquid over 12 h was recorded.

$$\begin{array}{c} \text{Sucrose preference}\;\left(\%\right)=\text{sucrose water consumption}/\\ \left(\text{sucrose water consumption}+\text{pure water consumption}\right)\times 100\% \end{array}$$

### 2.5 Gastric Emptying Rate and Small Intestinal Propulsion Rate

Five rats from each group were randomly selected to determine the gastric emptying and small intestinal propulsion rate. At the end of the treatment, rats were fasted without water for 24 h and then weighed. Based on their body weight, 2 mL/100 g of black semi‐solid paste (10 g sodium carboxymethyl cellulose, 16 g milk powder, 8 g starch, 8 g sucrose, 5 mL carbon ink dissolved in 250 mL pure water) was administered by gavage. 30 min later, 2% pentobarbital sodium (0.2 mL/100 g) was administered to anesthetize the rats. The stomach and small intestine were removed, dried with filter paper, and the total and net stomach weights were recorded.

$$\begin{array}{ccc} & & \text{Gastric emptying rate}\\ & & =[1-(\text{total gastric weight}-\text{net gastric weight})/\\ & & \text{weight of black semi - solid paste}]\times 100\%. \end{array}$$

$$\begin{array}{ccc} & & \;\text{Small intestinal propulsion rate}\\ & & =\left(\text{the propulsion length of the semi}-\text{solid nutrient paste}\right.\\ & & \left.\text{the total length of the small intestine}\right)\times 100\%. \end{array}$$

### 2.6 H&E Staining

After anesthesia, the brains, gastric antrum, and proximal duodenal tissues were rapidly excised from the rats, rinsed with pre‐cooled phosphate‐buffered saline (PBS), and immediately fixed in 4% paraformaldehyde for 48 h. The tissues were subsequently dehydrated through a graded ethanol series, cleared in xylene, embedded in paraffin, and sectioned into 4–5 µm‐thick slices using a microtome. The sections were dried in an oven, deparaffinized in xylene, and rehydrated through graded ethanol. They were then stained with hematoxylin for 3–5 min, differentiated and blued for several seconds, and counterstained with eosin for 2–3 min (Staining kit, G1005, Servicebio). Finally, the sections were dehydrated in absolute ethanol, cleared in xylene, mounted with neutral resin, and examined under a microscope (M8, Precipoint, Germany).

### 2.7 Nissl Staining

Paraffin‐embedded rat brain sections were deparaffinized in xylene and rehydrated through graded ethanol, then immersed in Nissl staining solution for 5 min (G1036, Servicebio). After rinsing with distilled water, excess water was removed, and the sections were dried in a 65°C oven for 4 h. The sections were subsequently cleared in xylene for 10 min, mounted with neutral resin, and observed under a microscope to evaluate the neuronal morphology and the distribution of Nissl bodies in the hippocampal CA1 and CA3 regions.

### 2.8 Transmission Electron Microscopy (TEM)

Fresh duodenal tissue samples were cut into 1 mm^3^ pieces and fixed in 2.5% glutaraldehyde, followed by post‐fixation in 1% osmium tetroxide. After dehydration through graded ethanol, the samples were embedded in epoxy resin. Ultrathin sections (60–80 nm) were prepared using an ultramicrotome, mounted on copper grids, and stained with uranyl acetate and lead citrate. The ultrastructural changes in the duodenal mucosa were then examined using transmission electron microscopy (HT7800; HITACHI, Japan).

### 2.9 Detection of Oxidative Stress Levels

Rat brain sections were incubated with 200 µL of fluorescent staining working solution (BB‐460513, BestBio) at 37°C for 50 min in the dark. After removal of the staining solution, the sections were incubated with wash buffer for 2 min and washed three times. The sections were then air‐dried, mounted, and observed under a confocal microscope (FV10i, Olympus, Japan) with an excitation wavelength of 488 nm and an emission wavelength of 525 nm. The relative fluorescence intensity was quantified using ImageJ software.

Fresh rat hippocampal tissue (100 mg) was placed in 900 µL of pre‐cooled PBS containing 1% protease inhibitor and homogenized using an ultrasonic homogenizer. The homogenate was then centrifuged at 4°C and 12,000 rpm for 20 min, and the supernatant was collected and kept on ice for subsequent analysis. Relevant biochemical indicators were measured using assay kits. Malondialdehyde (MDA) levels were determined using the thiobarbituric acid method (A003‐1, Nanjing Jiancheng). Blank and standard tubes were prepared, and the corresponding reagents were added according to the instructions. The reaction mixture was incubated in a 95°C water bath for 40 min, cooled under running water, and centrifuged at 4000 rpm for 10 min. Then, 200 µL of the supernatant was transferred to a microplate, and the absorbance was measured at 532 nm using a microplate reader. Superoxide dismutase (SOD) activity was determined using the hydroxylamine method (A001‐1‐1, Nanjing Jiancheng). Control tubes were prepared, and the corresponding reagents were added according to the kit protocol. The reaction mixture was incubated in a 37°C water bath for 40 min, followed by color development at room temperature in the dark for 10 min. Subsequently, 200 µL of the reaction solution was used for absorbance measurement at 550 nm. Three replicate wells were set for each sample. All procedures were performed strictly in accordance with the instructions.

### 2.10 JC‐1 Staining

Fresh hippocampal tissue (100 mg) was collected and washed with pre‐chilled PBS. Subsequently, 1 mL of Mitochondria Isolation Reagent was added, and the tissue was homogenized on ice. The homogenate was centrifuged at 1000 × *g* for 10 min at 4°C. The supernatant was then transferred and centrifuged at 12,000 × *g* for 10 min to pellet the mitochondria. The pellet was resuspended in 500 µL of Mitochondria Isolation Reagent and centrifuged again under the same conditions. After discarding the supernatant, the mitochondrial pellet was resuspended in 100 µL of Mitochondria Storage Solution (G3750‐50T, Servicebio). The mitochondrial suspension was mixed with JC‐1 staining working solution at a 1:9 ratio. The mixture was then observed under a confocal microscope to measure red and green fluorescence intensities. In this assay, red fluorescence indicates normal MMP, whereas green fluorescence reflects a decrease in MMP (G1515‐100T, Servicebio).

### 2.11 Immunofluorescence (IF)

Paraffin sections of the hippocampus and duodenum of rats were taken. After dewaxing and hydration, 0.01 mol/L sodium citrate antigen retrieval solution (pH 6.0) was used for 20 min of antigen retrieval. After naturally cooling to room temperature, the sections were washed three times with PBS and subsequently blocked with 5% BSA at room temperature for 1 h. Subsequently, the primary antibody Iba‐1 (1:300, 10904‐1‐AP; Proteintech) was added to the hippocampal tissue sections. Duodenal tissue sections were added with the primary antibody ZO‐1 (1:400, 21773‐1‐AP; Proteintech) and the primary antibody Occludin (1:400, 27260‐1‐AP; Proteintech), and incubated at 4°C overnight. The next day, they were washed three times with PBS, and fluorescently labeled secondary antibodies (1:300, goat anti‐rabbit IgG (H+L), A0516, A0562; Beyotime) were added. The sections were incubated at room temperature in the dark for 1 h, washed three times with PBS, and finally sealed with a quenching agent containing DAPI. Imaging was conducted under a confocal microscope, and the fluorescence expression region of the target protein was statistically analyzed using ImageJ software.

### 2.12 Western Blotting (WB)

Rat hippocampal tissue (100 mg) was collected, and total protein was extracted. Protein concentration was determined using the BCA method. After the addition of 5× loading buffer, the samples were heated at 95°C to denature the proteins. The proteins were then separated by 10% SDS‐PAGE, with 30 µg of protein loaded per lane, and subsequently transferred onto PVDF membranes for blocking. The membranes were incubated overnight at 4°C with the following primary antibodies: NLRP3 (1:1000, DF7438), Caspase‐1 (1:1000, AF5418), ASC (1:1000, DF6304), GSDMD (1:1000, AF4012), and β‐actin (1:10,000, AF7018), all purchased from Affinity. On the following day, the membranes were incubated with the secondary antibody (1:10,000, goat anti‐rabbit IgG HRP, S0001; Affinity) at room temperature for 1 h. ECL chemiluminescent substrate was then added, and images were captured within the linear exposure range using a chemiluminescence imaging system. Gray values were analyzed using ImageJ software, and the relative protein expression level was calculated as the ratio of the gray value of the target protein to that of β‐actin.

### 2.13 Enzyme‐Linked Immunosorbent Assay (ELISA)

After the rats were anesthetized, blood was collected from the abdominal aorta and placed in a centrifuge tube without anticoagulant. It was left to stand at room temperature for 1 h and then centrifuged for 15 min at 4°C and 3000 r/min. The upper serum was collected for the detection of the contents of Motilin (MTL) (AD1641Ra), Gastrin (GAS) (AD3309Ra), 5‐hydroxytryptamine (5‐HT) (AD2930Ra), IL‐6 (AD3249Ra), and tumor necrosis factor‐α (TNF‐α) (AD1723Ra). All kits were purchased from Andy Gene Company. Another 50 mg of hippocampal and duodenal tissue was weighed and added to PBS lysis buffer containing 1% protease inhibitor at a ratio of 1:9 and then thoroughly homogenized on ice. The homogenate was centrifuged for 20 min at 4°C and 12,000 r/min. The supernatant was collected to determine the contents of 5‐HT, IL‐6, and TNF ‐α in the hippocampus and duodenal tissues. All the above‐mentioned tests measured the absorbance values at a wavelength of 450 nm. The intra‐assay coefficient of variation was less than 9%, and the inter‐assay coefficient of variation was less than 11%. The detection range was 20 to 400 ng/ L. Eight replicate wells were set for each sample. All operations were strictly carried out in accordance with the instructions.

### 2.14 Quantitative Real‐Time Polymerase Chain Reaction (RT‐qPCR)

Rat hippocampal tissue (100 mg) was collected, and total RNA was extracted by the Trizol method. RNA concentration and purity (A260/A280 ratio between 1.8 and 2.0) were detected using a NanoDrop spectrophotometer. RNA was reverse transcribed into cDNA using a reverse transcription kit (CW2582M, CWBIO). Reaction conditions: 42°C for 15 min, 85°C for 5 min. Subsequently, cDNA amplification was carried out using the amplification kit (CW2601M, CWBIO), with a reaction system of 25 µL. Amplification procedure: pre‐denaturation at 95°C for 10 min, denaturation at 95°C for 15 s, and annealing and extension at 60°C for 1 min, totaling 40 cycles. Finally, dissolution curve analysis was conducted (95°C for 15 s, 60°C for 1 min, 95°C for 15 s, 60°C for 15 s). Three replicate wells were set for each sample, and the specificity was verified by a template‐free control. Taking β‐actin as the internal reference gene, the relative expression level was calculated by the 2^−△△Ct^ method. The primers were synthesized by Shanghai Shenggong Bioengineering Co., Ltd. All primers were verified before use. The primer sequence is shown in Table 1.

**TABLE 1:** Primer sequence.

Gene	Primer sequence (5′–3′)	Amplicon Size (bp)
NLRP3‐F NLRP3‐R	ACCCAAACCCACCAGTC CTCCCCTCACAGCCTTC	141
Caspase‐1‐F Caspase‐1‐R	CCCTCAAGTTTGCCCT CATCAGCTCCGACTCTCC	156
IL‐1β‐F IL‐1β‐R	AGTTGACGGACCCCAAA TCTTGTTGATGTGCTGCTG	147
IL‐18‐F IL‐18‐R	GGAGGGTTGTGTCCAG AATACAGGCGAGGTCATCA	100
β‐actin‐F β‐actin‐R	TCAGGTCATCACTATCGGCAAT AAAGAAAGGGTGTAAAACGCA	141

**TABLE 2:** Primer sequence information.

Type	Region of sequence	Primer names	Primer sequence	Length of product
16S bacteria	V3–V4	338F 806R	ACTCCTACGGGAGGCAGCA GGACTACHVGGGTWTCTAAT	∼466

### 2.15 16S RDNA Analysis of Intestinal Contents Samples

The total DNA of fecal samples was extracted using the OMEGA Soil DNA Kit (D5635‐02; Omega Bio‐Tek, Norcross, GA, USA) in accordance with the instructions of the kit. The integrity of the extracted DNA was detected by 0.8% agarose gel electrophoresis, and the concentration and purity of the DNA were measured by a NanoDrop micro‐spectrophotometer. Using total DNA as the template, the universal primers of 16S rRNA, 338F 5′‐ACTCCTACGGGAGGCAGCA‐3′) and 806R (5′‐GGACTACHVGGGTWTCTAAT‐3′), as shown in Table 2, were used to amplify the V3‐V4 regions of the gene. The PCR products were detected by 1% agarose gel electrophoresis. After confirming that the size of the target band met expectations, they were purified using a nucleic acid purification kit. The product was quantified using the Quant‐iT PicoGreen dsDNA Assay Kit. Sequencing libraries were constructed using the NEB Next Ultra II DNA Library Prep Kit (New England Biolabs, Inc., USA). After passing quality control, the library was sequenced at both ends on the Illumina NovaSeq 6000 platform (Illumina, Inc., USA). The OTU analysis process was adopted, and clustering was carried out based on 97% sequence similarity to obtain operational taxonomic units (OTUs). Species annotation was completed using the SILVA database, and the composition and relative abundance of microbiota at each taxonomic level were statistically analyzed. α‐Diversity analysis was used to evaluate the richness and diversity of the microbiota within the samples, and β‐diversity analysis was used to assess the structural differences of the microbiota among each group of samples. LEfSe analysis was used to screen the dominant microbiota with statistical significance, and the contribution degree was evaluated by LDA score.

### 2.16 Statistical Analysis

Statistical analysis was conducted using SPSS 24.0 software, and chart design was carried out using GraphPad Prism 9.5 software. The results that conform to the normal distribution are all expressed as mean ± SD. For comparisons between two groups, a two‐tailed independent sample *t*‐test was used, and one‐way analysis of variance (ANOVA) was used for comparisons among multiple groups. When the variances were uniform, the LSD test was used; when the variances were not uniform, the Tamhane's T2 test was used. For body weight data, repeated‐measures two‐way ANOVA was used for evaluation, followed by Tukey's multiple comparisons test for post hoc comparisons at individual time points. A *p*‐value <0.05 was considered statistically significant.

## 3 Results

### 3.1 The Effects of EA on Body Weight and Depression‐Like Behaviors in Rats

Weight changes and specific behavioral performance are key observational indicators for evaluating depressive states (Zou et al. 2023). In this study, the body weight changes of rats were monitored throughout the process, and the effects of EA treatment were evaluated by analyzing the results of the Open Field Test (OFT), Elevated Plus Maze (EPM), and SPT. It was found that the weight gain of rats in the control group was stable, while that of rats in the model group was significantly slower than that of the control group (Figure 1B). There was a significant difference in weight at the 10th week (Figure 1C; *p* < 0.0001), and EA treatment could significantly promote the increase in their body weight (Figure 1C; *p* < 0.0001). As shown in Figure 1D, compared with the control group, the sucrose preference index of rats in the model group decreased significantly (*p* < 0.0001). Compared with the model group, the sucrose preference rate in the EA group was significantly increased (*p* < 0.0001). Meanwhile, the results of OFT and EPM showed that the model group exhibited defects in autonomous activity and exploration behavior, with a significant reduction in total movement distance and central area stay time (*p* < 0.0001, *p* < 0.0001), and a significant decrease in the number of entries into the open arm and the proportion of stay time (*p* < 0.0001, *p* < 0.0001). After EA treatment, the total movement distance increased significantly (*p* < 0.0001), the stay time in the central area increased (*p* < 0.001), and the number of open arm entries and the proportion of stay time increased significantly (*p* < 0.0001, *p* < 0.0001). The results show that EA can effectively alleviate anhedonia and anxiety‐like behaviors in model rats and enhance their autonomous activity and exploration abilities.

### 3.2 The Effects of EA on Gastrointestinal Motility and Intestinal Barrier in Rats

In recent years, more and more studies have shown that the duodenum plays a key role in the mechanism of dyspepsia, and its impaired mucosal barrier integrity and low‐grade inflammation may ultimately lead to dyspepsia (Wauters et al. 2020). Transmission electron microscopy (TEM) (Figure 2A) showed that, compared with the control group, the duodenal mucosal epithelial cells in the model group were significantly damaged, as evidenced by increased cytoplasmic vacuolization, prominent mitochondrial swelling with disrupted cristae, reduced and disorganized microvilli with partial shedding, and indistinct intercellular junctions. Compared with the model group, the EA group showed reduced cytoplasmic vacuolization, attenuated mitochondrial swelling, a more regular arrangement of microvilli, clearer intercellular junctions, and a relatively intact overall epithelial structure. HE staining results showed (Figure 2B) that in the control group, the gastric tissue exhibited clear structural layers and orderly arrangement; the duodenal mucosal epithelial cells were tightly and neatly arranged, the villi were regular in shape and intact, the crypt structures were clear, and there was no inflammatory cell infiltration in the mucosal layer. In the model group, the gastric mucosal layer exhibited a disorganized and loose arrangement, while duodenal villi were disorganized, showing fusion and shedding. Compared with the control group, the number of eosinophils increased (*p* < 0.0001). Following EA treatment, the morphology of gastric tissue improved, with a more orderly arrangement, and the morphology of duodenal villi was improved, with a significant reduction in eosinophilic infiltration (*p* < 0.001). Gastric emptying rate and small intestinal propulsion rate are the core indicators reflecting the function of gastrointestinal motility (Zhang et al. 2022). The results showed (Figure 2C) that the gastric emptying rate and small intestinal propulsion rate were significantly reduced in the model group compared with the control group (*p* < 0.001, *p* < 0.0001), and the gastric emptying rate and small intestinal propulsion rate were increased after EA treatment (*p* < 0.001, *p* < 0.01). The results of serum gastrointestinal hormones showed (Figure 2D) that serum MTL and GAS levels were significantly decreased in the model group compared with the control group (*p* < 0.0001, *p* < 0.0001), and both levels were increased after EA intervention (*p* < 0.01, *p* < 0.01). IF detection further showed (Figures 2E–H) that the expression of tight junction proteins ZO‐1 and Occludin in the duodenum of the model group was downregulated compared with that of the control group (*p* < 0.001, *p* < 0.0001), suggesting that the structure of the tight junction was impaired and the intestinal mucosal barrier function was weakened, and the expression of ZO‐1 and Occludin proteins was restored to varying degrees after EA intervention (*p* < 0.01, *p* < 0.001), indicating that EA has a protective and repairing effect on the intestinal mucosal barrier.

**FIGURE 2:**
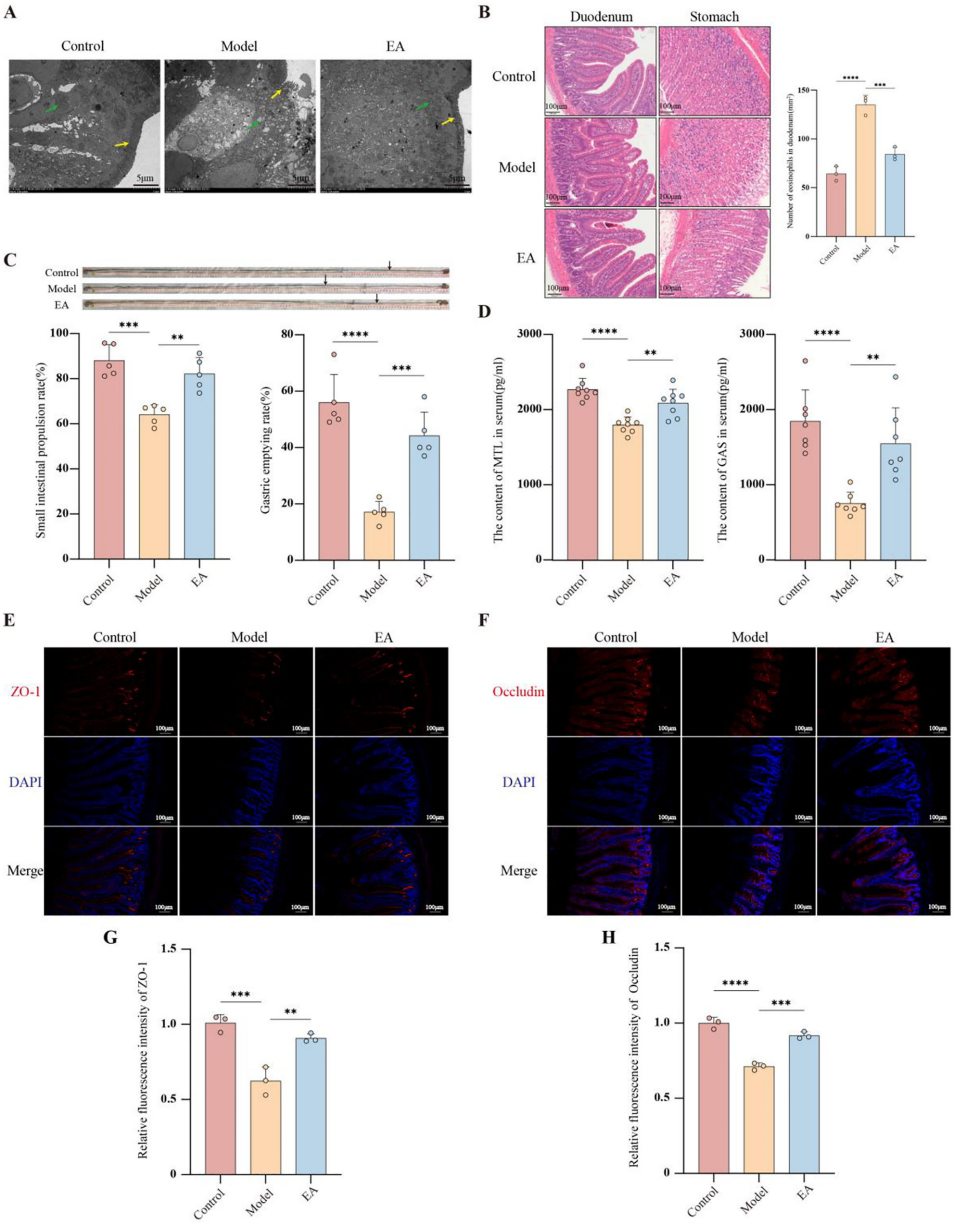
Effects of EA on dyspepsia and intestinal barrier in rats. (A) Observation of the duodenum by TEM. The yellow arrows indicate microvilli, and the green arrows indicate mitochondria. Scale bars = 5 µm. (B) HE staining of the duodenum and stomach, and the number of eosinophils in the duodenum (*n* = 3). Scale bars = 100 µm. (C) Small intestinal propulsion rate and gastric emptying rate assay; arrowheads indicate the termination of small intestinal propulsion (*n* = 5). (D) MTL and GAS assay (*n* = 8). (E–H) IF assay for duodenal tight junction protein ZO‐1 and Occludin protein expression (*n* = 3). Scale bars = 100 µm. Data are expressed as mean±SD. ^**^ *p* < 0.01, ^***^ *p* < 0.001, ^****^ *p* < 0.0001.

### 3.3 The Effects of EA on Gut Microbiota in Rats

We used 16S rRNA sequencing to evaluate the effects of EA on the gut microbiota in rats. The Venn diagram results (Figure 3A) showed partial overlap and significant differences in the species composition of the control group, the model group, and the EA group. To assess the species richness and diversity of samples from each group, we performed α‐diversity analysis using the Chao1 index and the Shannon index. The results showed (Figures 3B,C) that, compared with the control group, the Chao1 index was significantly elevated in the model group (*p* < 0.01); compared with the model group, the Chao1 index of the EA group decreased (*p* < 0.05). Regarding the Shannon index, compared with the control group, the Shannon index of the model group increased (*p* < 0.0001); after EA intervention, the Shannon index decreased (*p* < 0.01). These results indicate that the species richness of gut microbiota increased in model rats, while EA intervention reduced microbial diversity and may restore species richness to the control group. Community β‐diversity was assessed using principal coordinate analysis (PCoA) and non‐metric multidimensional scaling (NMDS) (Figure 3D,E). The results revealed distinct clustering between the model and control groups (Adonis: weighted UniFrac, *R* ^2^ = 0.6068, *p* = 0.007; Anosim: weighted UniFrac, *R* = 0.992, *p* = 0.006). After EA intervention, the microbial composition shifted closer to that of the control group (Adonis: weighted UniFrac, *R* ^2^ = 0.5313, *p* = 0.007; Anosim: weighted UniFrac, *R* = 0.96, *p* = 0.005). These results suggest that EA may improve gut microbiota structure in model rats, promoting its recovery toward the control group.

**FIGURE 3:**
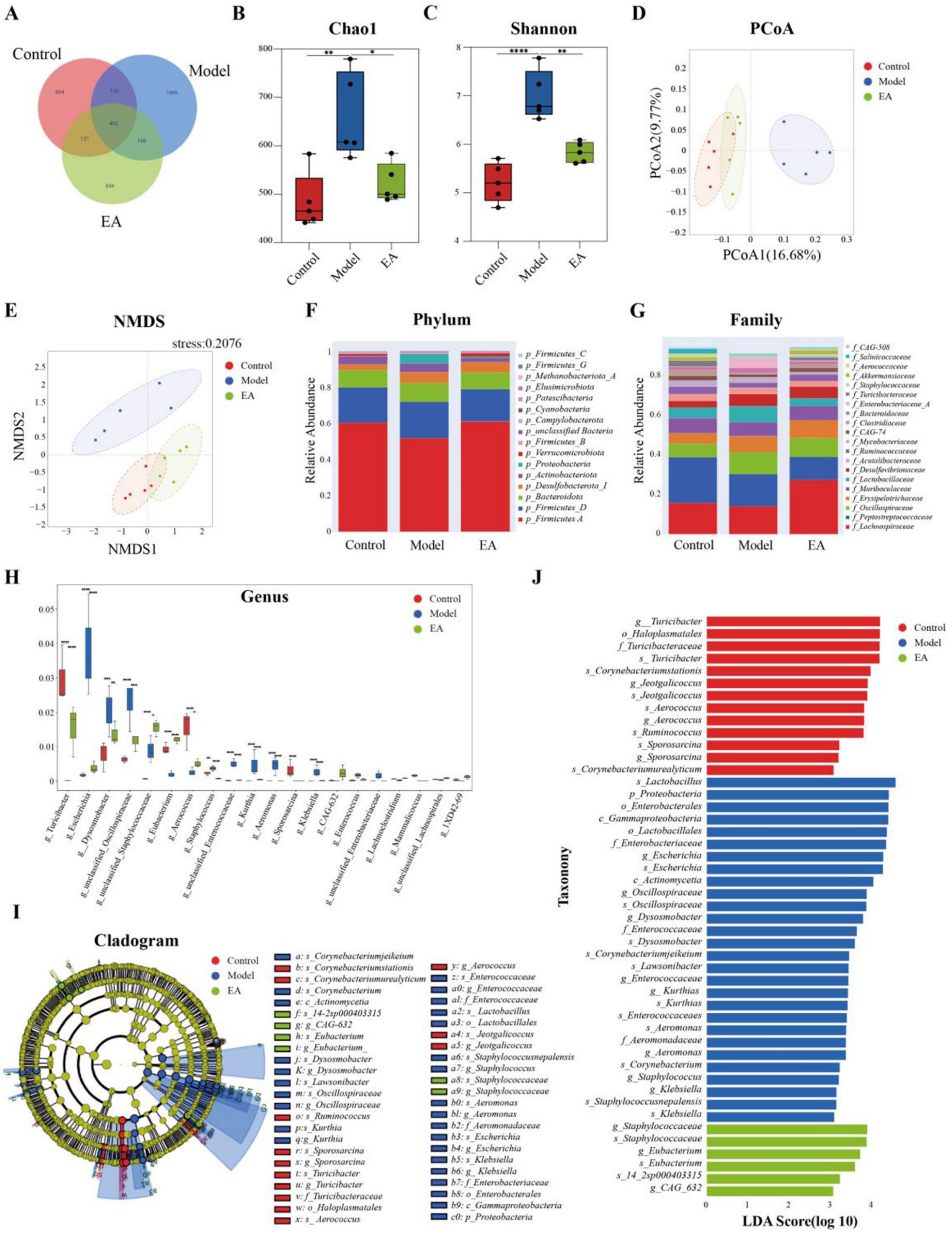
The effects of EA on the gut microbiota in rats. (A) Venn diagram of the gut microbiota. (B) Chao1 index. (C) Shannon index. (D) PCoA index. (E) NMDS index. (F) Relative abundance at the phylum level. (G) Relative abundance at the family level. (H) Relative abundance at the genus level. (I) Phylogenetic cladogram of gut microbiota in rats. (J) LDA score. Data are expressed as mean ± SD. ^*^ *p* < 0.05, ^**^ *p* < 0.01, ^***^ *p* < 0.001, ^****^ *p* < 0.0001. *n* = 5 per group.

To further analyze the effect of EA on gut microbial composition, we analyzed the relative abundance of taxa at the phylum, family, and genus levels among the three groups. At the phylum level (Figure 3F), the model group exhibited reduced abundances of Verrucomicrobia and Firmicutes and an increased abundance of Proteobacteria relative to the control group, whereas EA treatment markedly reversed these changes. At the family level (Figure 3G), compared with the control group, the model group showed significantly increased relative abundances of Enterobacteriaceae, Oscillospiraceae, and Lactobacillaceae, while the relative abundances of Akkermansiaceae and Turicibacteraceae were significantly decreased. These alterations were reversed after EA treatment. We screened for bacterial genera that showed trends of differential abundance among the three groups at the genus level (Figure 3H). Compared with the control group, the model group showed significantly decreased abundances of Turicibacter and Eubacterium and increased abundances of Escherichia and Dysosmobacter (*p* < 0.001), and these changes were reversed by EA treatment (*p* < 0.01).

LEfSe analysis results (Figures 3I–J) showed that dominant taxa in the control group included Turicibacter and Aerococcus. In the model group, Escherichia, Enterococcaceae, and Oscillospiraceae were identified as dominant taxa. In the EA group, Eubacterium was the predominant taxon. In summary, model rats exhibited gut microbiota dysbiosis, whereas EA effectively ameliorated microbial imbalance and shifted the overall microbial composition toward that of the control group.

### 3.4 The Effect of EA on the mtROS‐NLRP3 Pathway and Microglial Activation in the Hippocampus of Rats

The results of HE staining showed that in the control group of rats, the neurons in the hippocampal CA1 area and CA3 area were closely and neatly arranged. In the model group, the pyramidal cell layers in the CA1 and CA3 areas of the hippocampus were loosely arranged, the cell bodies shrank, and some cells showed cytoplasmic vacuolar degeneration and nuclear consolidation. After EA treatment, the above pathological changes were alleviated; the neurons were closely arranged, and the cell morphology was significantly improved (Figure 4A). The Nissl staining results showed that in the control group of rats, the neurons in the CA3 and CA1 regions of the hippocampus were closely arranged, the number of Nissl bodies was abundant, and the cell morphology was intact. In the model group, the arrangement of neurons in the CA3 area was disordered and significantly loose. Compared with the control group, the number of Nissl bodies in the CA3 and CA1 regions decreased (*p* < 0.01), suggesting damage to hippocampal neurons. After EA treatment, the arrangement of neurons was restored, and the number of Nissl bodies increased (*p* < 0.05), indicating that EA has an improving effect on hippocampal neuron injury (Figure 4B).

**FIGURE 4:**
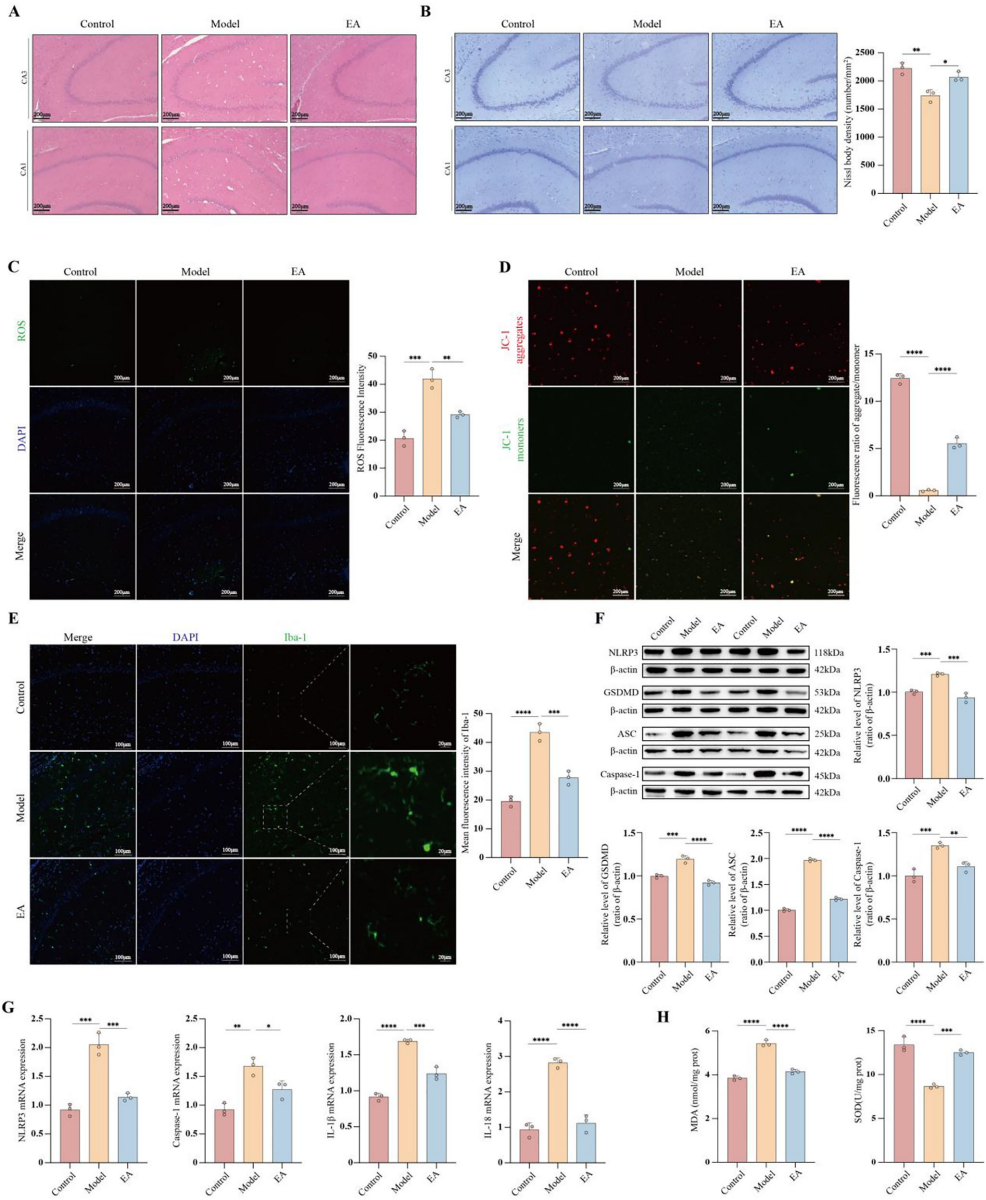
Effects of EA on hippocampal neuroinflammation in rats. (A) HE staining of hippocampal CA3 and CA1 regions. Scale bars = 200 µm. (B) Nissl staining of hippocampal CA3 and CA1 regions. Scale bars = 200 µm. (C) ROS levels in the hippocampus. Scale bars = 200 µm. (D) JC‐1 assay for MMP in the hippocampus. Scale bars = 200 µm. (E) IF detection of Iba‐1 expression in the hippocampus. Scale bars = 100 µm. (F) Western blot detection of hippocampal NLRP3, ASC, caspase‐1, and GSDMD protein expression. (G) RT‐qPCR detection of hippocampal NLRP3, caspase‐1, IL‐1β, and IL‐18 mRNA expression. (H) Biochemical detection of hippocampal MDA and SOD levels. Data are expressed as mean ± SD. ^*^ *p* < 0.05, ^**^ *p* < 0.01, ^***^ *p* < 0.001, ^****^ *p* < 0.0001. *n* = 3 per group.

The results in Figures 4C,D showed that the ROS fluorescence signal in the CA1 region of the hippocampus of the model group was enhanced compared with that of the control group (*p* < 0.001), and the mitochondrial red/green fluorescence intensity ratio of the hippocampus decreased dramatically (*p* < 0.0001), whereas after EA treatment, the ROS fluorescence intensity was significantly attenuated (*p* < 0.01), and the hippocampal mitochondrial red/green fluorescence intensity ratio increased (*p* < 0.0001). The above results indicated that EA effectively reduced hippocampal mtROS levels, stabilized MMP, and attenuated mitochondrial dysfunction. Meanwhile, the results of the biochemical assay showed (Figure 4H) that the MDA content of the hippocampus in the model group was significantly elevated (*p* < 0.0001), and the SOD activity was reduced (*p* < 0.0001); after EA treatment, the MDA content was reduced (*p* < 0.0001), and the SOD activity was elevated (*p* < 0.001), which further verified that EA could alleviate hippocampal oxidative stress injury.

IF results showed (Figure 4E) that, compared with the control group, the model group exhibited increased mean Iba‐1 fluorescence intensity in the hippocampal CA1 region (*p* < 0.0001); microglia showed enlarged cell bodies and reduced branching, indicating an activated state. After EA treatment, the mean Iba‐1 fluorescence intensity decreased (*p* < 0.001), and microglial cell bodies became smaller, suggesting a shift toward the resting state. Western blot results showed (Figure 4F) that, compared with the control group, the protein expression levels of NLRP3, ASC, Caspase‐1, and GSDMD in the hippocampus of the model group were upregulated (all *p* < 0.001); after EA treatment, the expression levels of the above proteins decreased (all *p* < 0.01). In addition, NLRP3, Caspase‐1, IL‐1β, and IL‐18 mRNA expression was elevated in the hippocampus of the model group (all *p* < 0.01); after EA treatment, the mRNA expression of the above genes was reduced (all *p* < 0.05) (Figure 4G).

### 3.5 The Effects of EA on Central and Peripheral 5‐HT and Inflammatory Factors in Rats

As a core signaling molecule in the bidirectional regulation of the brain–gut axis, the imbalance in the distribution and function of 5‐HT is a key link between central neuroinflammation and peripheral gastrointestinal inflammation (Qu et al. 2024). Our results showed that compared with the control group, the expression of 5‐HT in the hippocampus, serum, and duodenum was significantly downregulated in the model group of rats (all *p* < 0.0001), accompanied by increased levels of TNF‐α and IL‐6 (all *p* < 0.0001). After EA intervention, 5‐HT levels in the hippocampus, serum, and duodenum of rats were upregulated compared with the model group (all *p* < 0.001), accompanied by decreased levels of TNF‐α and IL‐6 (all *p* < 0.01). (Figure 5A–C). These results suggest that EA may modulate both central neuroinflammation and peripheral gastrointestinal inflammation, accompanied by increased 5‐HT levels in the central and peripheral systems and reduced TNF‐α and IL‐6 release.

**FIGURE 5:**
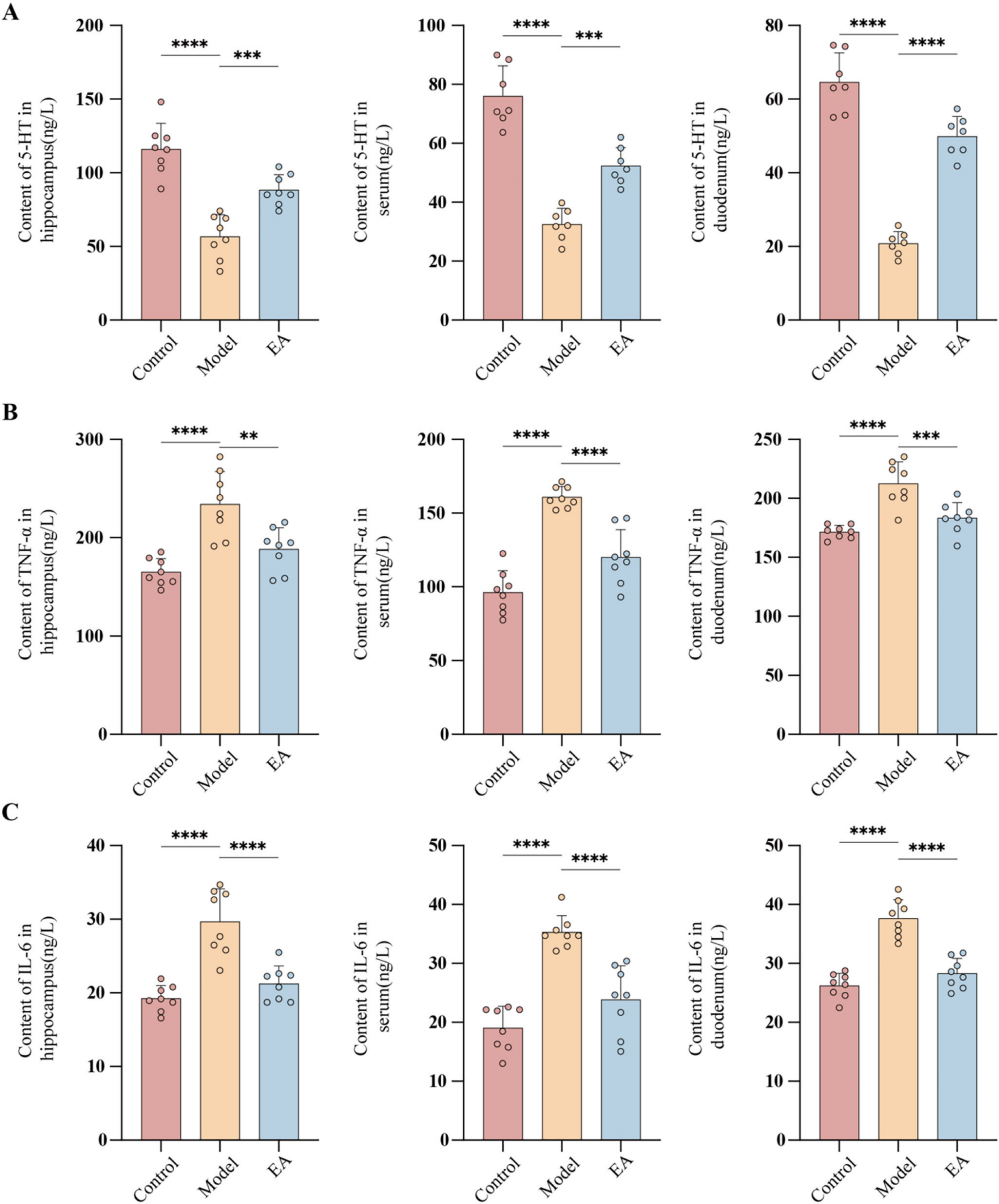
Effects of EA on 5‐HT, TNF‐α, and IL‐6 in rat hippocampus, serum, and duodenum. (A) Expression of 5‐HT in hippocampus, serum, and duodenum. (B) Expression of TNF‐α in hippocampus, serum, and duodenum. (C) Expression of IL‐6 in hippocampus, serum, and duodenum. Data are expressed as mean ± SD. ^**^ *p* < 0.01, ^***^ *p* < 0.001, ^****^ *p* < 0.0001. *n* = 8 per group.

## 4 Discussion

In this study, we evaluated the intervention effects of EA on behavioral abnormalities and gastrointestinal dysfunction in comorbid rats. The results showed that EA “ST36” and “LR3” could improve depression‐like behaviors and dyspepsia‐like symptoms in model rats. These improvements were accompanied by changes in central and peripheral 5‐HT levels, inflammatory markers, hippocampal oxidative stress and microglial/NLRP3‐related indices, gut microbial composition, and duodenal barrier‐related indicators (Figure 6).

**FIGURE 6:**
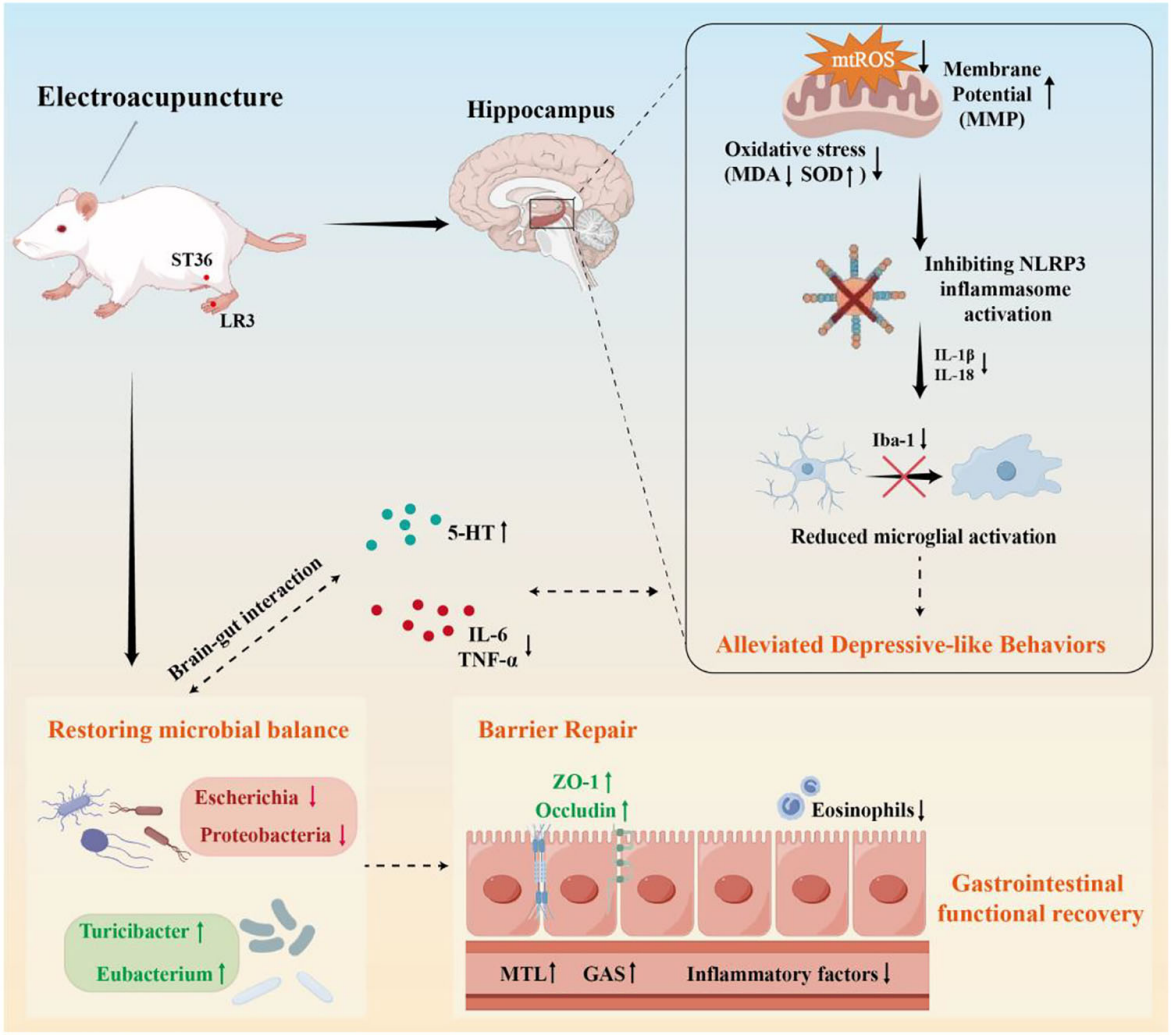
Hypothesis.

One of the core characteristics of depression is behavioral abnormality, specifically manifested as anhedonia, reduced autonomous activity, decreased exploratory behavior, increased anxiety‐like behavior, etc. (Liu et al. 2025). The results of this study showed that model rats exhibited decreased sucrose preference, along with reduced autonomous activity and exploratory behavior, which is consistent with previous studies in depression‐like models (Pei et al. 2025). After EA intervention, the sucrose consumption of rats increased, their autonomous activity and exploratory behavior increased, and anxiety‐like behaviors decreased, indicating that EA can alleviate depression‐like behaviors in rats. At the gastrointestinal function level, the model rats showed significant gastrointestinal motility deficiency, with decreased gastric emptying rate and small intestinal propulsion rate, and the levels of key hormones related to gastrointestinal motility (MTL and GAS) also decreased. One study found that gastrointestinal hormone levels change in the state of depression and can also be used as an indicator to predict the degree of depression (Yang et al. 2023). EA intervention can reverse the above abnormalities, suggesting that EA can effectively improve depressive states and gastrointestinal motility disorders. Recent studies have confirmed that damage to the duodenal mucosal barrier and low‐grade inflammation are key pathological mechanisms for the occurrence and development of dyspepsia (Puthanmadhom et al. 2022; Wauters et al. 2020). When the intestinal mucosal barrier is damaged, harmful substances in the intestinal lumen (such as lipopolysaccharides, food antigens, etc.) invade the lamina propria of the mucosa, activate immune cells, and trigger inflammatory responses. The released inflammatory factors can not only cause intestinal nerve sensitization but also send abnormal signals to the central nervous system, further aggravating intestinal motility disorders and eventually presenting as core symptoms of dyspepsia such as upper abdominal distension and pain (Miwa et al. 2019). Our study found that EA improved gastrointestinal histomorphology, reduced the expression levels of the inflammatory factors IL‐6 and TNF‐α in the duodenum, decreased eosinophil infiltration, ameliorated the ultrastructural damage of duodenal tissue, and upregulated the expression of the tight junction proteins ZO‐1 and Occludin. These findings suggest that EA may alleviate duodenal inflammation and contribute to the restoration of intestinal mucosal barrier function.

The hippocampus, as a key brain region for stress regulation and emotion integration in the central nervous system, has structural and functional abnormalities that are an important pathological basis for the occurrence of depression. Studies have confirmed that patients with depression often have reduced hippocampal volume, decreased neuronal number, and impaired prominent plasticity, which are closely related to the development of depressive symptoms (Fujii et al. 2022; Joshi et al. 2016). CA1 and CA3, as core subregions of the hippocampus, have structural integrity and plasticity that are particularly crucial in the process of depression. This study found that EA intervention significantly increased the thickness of the CA1 and CA3 cell layers, improved cell arrangement, and increased the number of Nissl bodies. These results suggest that EA may exert antidepressant effects by enhancing hippocampal neuroplasticity in the CA1 and CA3 regions. The hippocampus is highly sensitive to stress. Persistent stress can lead to elevated levels of glucocorticoids. High levels of glucocorticoids directly act on the mitochondrial membrane of hippocampal neurons, resulting in mitochondrial energy metabolism disorders (Ciubuc‐Batcu et al. 2024). Clinical studies have pointed out that depression is associated with mitochondrial dysfunction and enhanced related oxidative stress, and patients often present with decreased levels of antioxidant enzymes (Khan et al. 2023). In this study, model rats showed a significant decrease in hippocampal MMP, increased levels of ROS and MDA, and a declining trend in SOD activity. EA intervention reversed these alterations, suggesting that EA may exert its antidepressant effects by improving hippocampal mitochondrial function and attenuating oxidative stress injury. During the occurrence and development of depression, the oxidative stress state of hippocampal neurons directly triggers neuroinflammatory responses, thereby exacerbating depressive symptoms. A study shows that minocycline can effectively reduce hippocampal neuroinflammation by inhibiting mitochondrial oxidative damage and achieve the effect of relieving depression (Mozafari et al. 2020). As central immune cells and neuroinflammation regulators, microglia are closely associated with depression (Wang et al. 2022). This study found that EA could downregulate the expression of the microglial marker Iba‐1 in model rats and inhibit the expression of NLRP3 pathway‐related proteins and genes, suggesting that EA can alleviate neuroinflammation. Furthermore, a study found that quercetin inhibits microglial activation by promoting mitochondrial autophagy, reducing mtROS accumulation, and alleviating NLRP3 inflammasome activation (Han et al. 2021), which is consistent with the results of our study.

Hippocampal neuroinflammation can skew tryptophan metabolism away from 5‐HT synthesis toward kynurenine production, leading to decreased 5‐HT level. 5‐HT is a critical regulator of hippocampal neuroplasticity; its decline attenuates synaptic connectivity and suppresses hippocampal neurogenesis. One study found that restoration of tryptophan–kynurenine metabolic homeostasis attenuates neuroinflammation, suppresses hippocampal microglial proliferation, and ameliorates depression‐like behaviors in rats (Yao et al. 2025). Our study found that EA could increase the level of 5‐HT in the hippocampus of model rats, suggesting that it may exert antidepressant effects by modulating tryptophan metabolism. In addition, 5‐HT is a core neurotransmitter for bidirectional regulation of the brain–gut axis, and its expression changes in the periphery are closely related to gastrointestinal function. We found that after EA intervention, the levels of 5‐HT in the serum and duodenum of rats increased. These results suggest that the ameliorative effects of EA on depression‐like behaviors and the accompanying gastrointestinal dysfunction may be associated with its modulatory action on central and peripheral 5‐HT levels.

Previous studies have confirmed that neuroinflammation mediated by excessive activation of microglia, combined with 5‐HT signaling disorder, can affect the structure of the gut microbiota (Ma et al. 2023). Gut microbiota dysbiosis has been reported to affect the synthesis and release of mood‐related neurotransmitters and to amplify inflammatory responses, thereby potentially contributing to both depression progression and gastrointestinal dysfunction (Chen et al. 2025; Li et al. 2026). In our study, the gut microbial composition of rats after EA intervention tended to resemble that of the control group, suggesting that EA may partially restore stress‐related gut microbiota dysbiosis. Further analysis showed that EA significantly reduced the abundance of Proteobacteria, with Escherichia showing the most pronounced change. As an opportunistic pathogen, *Escherichia* overgrowth has been implicated in the onset and exacerbation of depression (Yun et al. 2020), and its reduction may help improve intestinal barrier integrity, alleviate gastrointestinal dysfunction, and maintain intestinal homeostasis (Zhang et al. 2025). After EA treatment, the abundance of Akkermansia was upregulated. This bacterium is closely related to intestinal barrier function and can promote the expression of intestinal tight junction proteins, which is consistent with the results of a previous study (Chen et al. 2024). In addition, the abundance of Turicibacter and Eubacterium increased after EA intervention. Turicibacter has been identified as an important genus involved in gut neuroendocrine signaling and 5‐HT homeostasis and is negatively associated with depression (Sun et al. 2022). Eubacterium has the ability to produce short‐chain fatty acids (SCFAs) (especially butyrate). Butyrate not only provides energy for intestinal epithelial cells but also enhances intestinal barrier function (Gasaly et al. 2021). Our results also revealed that EA downregulated the abundance of Enterococcaceae, Oscillospiraceae, and Dysosmobacter. It has been reported that an increase in Enterococcaceae aggravates the occurrence of depression‐like behaviors and intestinal inflammation (Jang et al. 2022), which is consistent with the results of this study; Oscillospiraceae and Dysosmobacter are regarded as SCFA‐producing bacteria in the intestinal tract and are generally considered beneficial bacteria. Several studies have found that promoting the enrichment of both can regulate the level of SCFAs to exert an antidepressant effect, which contradicts our results (Barandouzi et al. 2020; Zhu et al. 2025). It is notable that SCFAs can inhibit gastrointestinal motility and delay gastric emptying under certain conditions (Chen et al. 2020). We speculate that the increased abundance of Oscillospiraceae and Dysosmobacter in model rats may be a compensatory response to chronic stress and intestinal inflammation and may potentially be linked to dyspepsia‐related symptoms. However, this interpretation remains speculative, as SCFA levels were not measured and no functional microbiome analyses were performed in the present study. Further metabolomic and mechanistic studies are needed to directly test this possibility.

Our research still has certain limitations. Firstly, this study did not include an iodoacetamide‐only group or a CUMS‐only group; therefore, the individual effects of these two modeling procedures and their combined effects could not be evaluated separately. Secondly, although the results support that the mtROS/NLRP3 pathway is closely related to the efficacy of EA, this pathway has not yet been specifically blocked or activated through NLRP3 inhibitors, mtROS clearance agents, and other means. Therefore, the explanation of the causal relationship still needs to be further strengthened. Thirdly, although this study observed concurrent changes in neuroinflammation, 5‐HT levels, and gut microbiota composition, these indicators were measured in parallel. Therefore, it is not possible to infer a direct interaction between neuroinflammation and gut microbiota, nor to prove that 5‐HT mediates microbial remodeling. Meanwhile, this study also failed to elucidate the metabolic functions of specific microbial taxa or the precise mechanisms underlying their interactions with neuroinflammation. The relatively small sample size in each group may have limited statistical power and result stability. Future studies incorporating larger sample sizes, fecal microbiota transplantation, microbiota depletion, metagenomic sequencing, metabolomics, and interventions targeting 5‐HT‐related pathways are needed to further elucidate the causal relationships and underlying mechanisms.

## 5 Conclusion

In summary, this study found that EA “ST36” and “LR3” could significantly improve the depression‐like behaviors and dyspepsia‐like symptoms in comorbid rats. These improvements were accompanied by reduced hippocampal oxidative stress and microglial/NLRP3‐related inflammatory responses, changes in central and peripheral 5‐HT levels, alterations in gut microbial composition, and improved duodenal barrier‐related indices. These findings suggest that the therapeutic effects of EA may be associated with coordinated changes in the brain–gut axis; however, this study was unable to determine the causal relationship between the cerebral and intestinal changes, and the specific mechanisms still require further in‐depth investigation.

## Author Contributions

**Tianlang Wang**: writing – original draft. **Yifan Feng**: formal analysis. **Xihan Wang**: software. **Yanan Xue**: methodology. **Jianbo Wang**: methodology. **Yi Qu**: conceptualization. **Tieming Ma**: writing – review and editing. **Jidong Liu**: writing – review and editing, funding acquisition. All authors have read and approved the final version of the manuscript.

## Funding

This study was supported by the National Natural Science Foundation of China (NO.82474634) and Liaoning Provincial Science and Technology Program Joint Program (2024JH2/102600199).

## Ethics Statement

This study was conducted in accordance with the relevant animal ethical standards, and the rats were purchased from Liaoning Changsheng Biotechnology Co., Ltd, license number (SCXK (Liao) 2020‐0001), and this study has been approved by the Ethical Review and Approval of the Center for Animal Experimentation, Liaoning University of Traditional Chinese Medicine (Ethical Review Approval Number: 21000062024086).

## Conflicts of Interest

The authors declare no conflicts of interest.

## Data Availability

Data will be made available on request.
